# Home deliveries and Hydrocolpos: A Rare Case Presentation of Imperforate Hymen Diagnosed at 4 Months of Age

**DOI:** 10.51894/001c.144431

**Published:** 2025-09-30

**Authors:** Alexander Benben

**Affiliations:** 1 Department of Urology Detroit Medical Center

## 69

### INTRODUCTION

Imperforate hymen, a rare congenital anomaly of the female genitourinary tract, is defined as the hymen completely obstructing the vaginal opening. We present an unusual case of imperforate hymen in a 4 month of child causing hydrocolpos and severe bilateral hydroureteronephrosis.

### CASE DESCRIPTION

A 4 month old female presented to the emergency department with worsening abdominal distension and decreased urine output. Abdominal imaging revealed a large cystic mass within the pelvis and bilateral hydroureteronephrosis with debris in the collecting system (Figure with imaging to be included). Her past medical history was significant for a home midwife delivery and no prior examination by a pediatrician. Her family reported normal antenatal ultrasounds, although it was unclear when the final ultrasound was performed. She was on no medications, however, her parents did apply topical essential oils. Initial general surgery recommendations were for MRI. Pediatric urology noted a bulging, blue imperforate hymen on physical examination and took her urgently to the operating room. Incision of the hymen immediate returned 400 mL of thick milky fluid. A Foley catheter and Penrose drain were left in place overnight. At two month follow up, her hydronephrosis had resolved and she was thriving.

### DISCUSSION/CONCLUSIONS

A careful newborn examination is crucial to identify congenital anomalies that may have significant sequelae. Several diagnoses that cause hydrocolpos and hydronephrosis can be identified on physical examination, occasionally negating the need for extensive radiographic evaluation.

